# Epigallocatechin-3-Gallate Reduces Cd-Induced Developmental Toxicity of Bodysize in *Caenorhabditis elegans* via the PEK-1/eIF-2α/ATF-4 Pathway

**DOI:** 10.3390/molecules28176344

**Published:** 2023-08-30

**Authors:** Shuanghui Wang, Chuhong Chen, Yan Lu

**Affiliations:** 1 National Research Center of Engineering and Technology for Utilization of Botanical Functional Ingredients from Botanicals, Hunan Agricultural University, Changsha 410128, China; 2 Key Laboratory of Green Control of Crop Pests in Hunan Higher Education, Hunan University of Humanities Science and Technology, Loudi 417000, China

**Keywords:** tea, EGCG, cadmium, *Caenorhabditis elegans*, developmental toxicity, endoplasmic reticulum stress

## Abstract

Cadmium (Cd), a harmful heavy metal that has no biological purpose, can harm healthy fetal and child development. Epigallocatechin-3-gallate (EGCG), the most abundant polyphenol in tea, has been shown to increase cell viability under Cd exposure and ameliorate Cd-induced kidney injury in adult male rats. Using the *Caenorhabditis elegans* (*C. elegans*) model, we demonstrated that EGCG mitigated Cd-induced body size developmental toxicity through a mechanism that did not involve chelation with EGCG and was not associated with Cd accumulation and efflux. Our research indicated that the beneficial effects of EGCG on Cd-induced body size developmental toxicity were associated with the mitigation of endoplasmic reticulum stress. Furthermore, our observations indicate that EGCG reduced Cd-induced developmental toxicity in *C. elegans* via the PEK-1/eIF-2α/ATF-4 pathway. Our results provide important evidence for the potential benefits of consuming tea as a detoxification agent.

## 1. Introduction

Cadmium (Cd), a harmful heavy metal that has no biological purpose [1], can enter the body through eating contaminated food, smoking, or breathing contaminated air [2]. Once absorbed, Cd has the potential to accumulate in various tissues, including the kidneys, liver, testes, and ovaries, thereby increasing the risk of diseases such as chronic kidney disease, liver disease, and infertility [3].

Cd exposure occurs from the beginning of life because of its ability to cross the placental barrier [4], adversely affecting the healthy development of fetuses and children [5]. Exposure to Cd during pregnancy, both before and after birth, has been linked to various negative outcomes for the pregnancy, such as deformities, a decrease in the rate of fetal growth, lower birth weight, and mortality [6,7]. In addition, Cd exposure has been demonstrated to have developmental toxic effects across various animal models. For example, it retards larval development and reduces body length in *C. elegans* [8,9]; alters larval body size and prolongs pupation in *Drosophila* [10]; and causes disturbances in steroid hormone levels, leading to delayed onset of puberty and reduced ovarian follicle pool and formation in rats [11].

Tea is one of the most popular functional beverages in the world today, with a rich history dating back to ancient times [12]. According to the earliest surviving monograph on traditional Chinese medicine, “Shen Nong Materia Medica”, tea was originally consumed for its detoxifying effects. Tea can reduce the toxicity caused by various environmental toxicants, such as pesticides, smoke, mycotoxins, and arsenic [13]. Epigallocatechin-3-gallate (EGCG), which accounts for 50–75% of the total catechins in green tea polyphenols, is the most abundant polyphenolic compound in tea [12]. It is considered the most promising bioactive compound in green tea due to its potent antioxidant properties [14]. It has been shown to have ameliorative effects on a wide range of chronic diseases such as cardiovascular disease, cancer, and neurodegenerative diseases [12]. It can reduce the toxicity caused by pesticides such as paraquat [15], dichlorodiphenyltrichloroethane (DDT) [16], and methyl parathion [17]. It has been shown to increase the viability of HL-7702 and PC12 cells under Cd exposure and ameliorate Cd-induced renal injury in adult male Wistar albino rats [18,19,20]. However, it has also been shown that EGCG increases the toxicity of Cd to PC12 cells [21]. Therefore, whether EGCG decreases or increases Cd toxicity needs to be further investigated. In addition, there is currently no reported evidence on whether EGCG can reduce the body size developmental toxicity caused by Cd exposure.

*C. elegans* has the advantages of easy culture, short life cycle, small size, and transparent body, making it an ideal model organism for assessing the toxicity of environmental contaminants [22]. Body size is an important indicator of nematode growth and development and is easy to measure [23]. In this study, we investigated the effect of EGCG on Cd-induced somatic developmental toxicity in nematodes using *C. elegans* as a model system, and we analyzed the mechanism from the perspective of endoplasmic reticulum (ER) stress, providing important evidence for the detoxifying potential of tea.

## 2. Result

### 2.1. EGCG Alleviates Cd-Induced Body Size Developmental Toxicity in C. elegans

**Figure 1:**
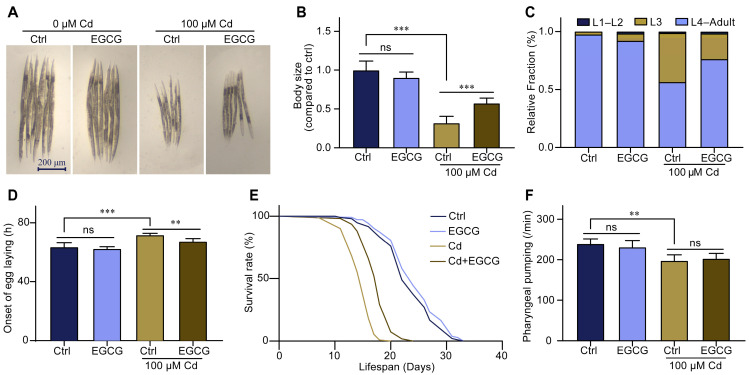
Epigallocatechin-3-gallate (EGCG) reduced the developmental toxicity of Cd-induced *C. elegans*. (**A**) Micrographs of nematodes treated with 100 μM Cd or/and 400 μM EGCG for 3 d from larval stage L1. (**B**) Body size of the nematodes treated with 100 μM Cd or/and 400 μM EGCG for 3 d from larval stage L1. Body size was normalized to the control without Cd or EGCG treatment. (**C**) Quantification of different developmental stages of nematodes treated for 48 h from L1 with 100 μM Cd or/and 400 μM EGCG. (**D**) The egg-laying onset was measured by intervention at 48 h with 100 μM Cd or/and 400 μM EGCG. (**E**) Survival curve intervention at 3 days from L1 with 100 μM Cd or/and 400 μM EGCG. (**F**) Pharyngeal pumping intervention at 3 days from L1 with 100 μM Cd or/and 400 μM EGCG, *n* ≥ 30. One-way ANOVA and Tukey post hoc tests were used to assess significance: ** *p* < 0.01, *** *p* < 0.001, ns = no significance.

In a Cd-free environment, low exposure concentrations of EGCG (≤400 μM) had no effect on nematode body size, whereas high exposure concentrations of EGCG (≥1000 μM) inhibited the development of nematode body size (Figure S1A,B). However, in a 100 μM Cd environment, EGCG partially restored the Cd-induced reduction in nematode body size. Specifically, body size exhibited a positive correlation with EGCG exposure concentrations in the range of 0–400 μM but showed no significant change at exposure concentrations of 400–1000 μM (Figure S1C,D). Thus, further experiments were carried out using 100 μM Cd and 400 μM EGCG (Figure 1A,B).

To further investigate the effect of EGCG on nematode development under Cd exposure, we determined the effect of EGCG on larval stage distribution and egg-laying onset. In the Cd-free environment, almost all nematodes developed normally to the L4 stage after 48 h with or without the presence of EGCG. In the 100 μM Cd environment, approximately half of the nematodes remained at the L3 stage; however, the proportion of L3 larvae was significantly increased by supplementation with EGCG (Figure 1C). The experiment on the egg-laying onset time showed that exposure to 100 μM Cd significantly prolonged the egg-laying onset time, while EGCG partially restored it (Figure 1D). The early developmental stage has a profound effect on individual senescence [24], and to confirm whether EGCG intervention at the larval stage improves lifespan, we determined the lifespan of nematodes that received intervention only at the larval stage. The findings demonstrated that exposure to EGCG, even at a concentration of 400 μM, did not affect nematode lifespan in a Cd-free environment but significantly prolonged it under Cd exposure (Figure 1E). Since food chewing can influence nematode development, we determined the pharyngeal pumping rate of nematodes to confirm that any observed effect of EGCG on nematode development under Cd exposure was not related to food intake. The results showed that EGCG had no significant effect on the pharyngeal pumping rate of nematodes; however, pharyngeal pumping rate was significantly reduced by exposure to Cd, and supplementation with EGCG did not increase the pharyngeal pumping rate (Figure 1F). In conclusion, our results show that EGCG ameliorates Cd-induced developmental toxicity in *C. elegans* independently of food chewing.

### 2.2. Detoxification of Body Size Developmental Toxicity by EGCG Is Not Related to Its Chelating Effect

**Figure 2:**
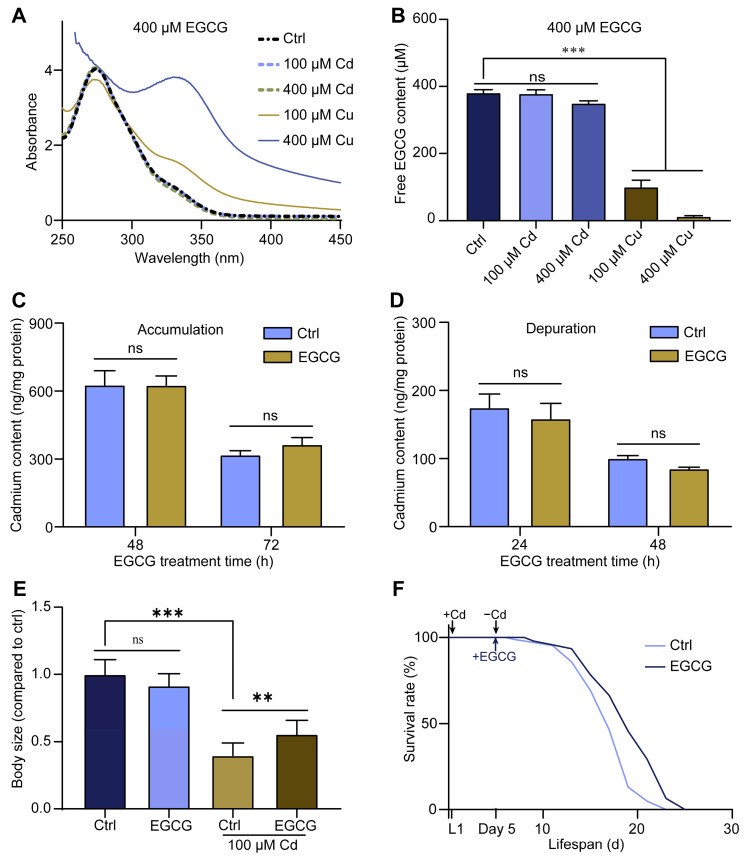
Detoxification of body size developmental toxicity by EGCG is not related to its chelating effect. (**A**) UV-Vis spectra of 400 μM EGCG treated with 100 or 400 μM Cd and Cu for 4 h in M9 medium. (**B**) Free EGCG content of 400 μM EGCG treated with 100 or 400 μM Cd and Cu for 4 h in M9 medium measured by HPLC. (**C**) The accumulation of Cd treated in *C. elegans* with EGCG (0 or 400 μM) was measured using ICP-MS. (**D**) The Cd content in L1 nematodes treated first with 100 μM Cd for 48 h and then with EGCG (0 or 400 μM) for 24 or 48 h. (**E**) Body size of L1 nematodes treated with Cd (0 or 100 μM) for 48 h and then with EGCG (0 or 400 μM) for 48 h. Body size was normalized to the control without Cd or EGCG treatment. (**F**) Survival curves of L1-stage wild type (WT) nematodes treated first with 100 μM Cd for 5 d and then with EGCG (0 or 400 μM). One-way ANOVA and Tukey post hoc tests were used to assess significance: ** *p* < 0.01, *** *p* < 0.001, ns = no significance.

EGCG is a metal ion chelator that can chelate Cd, thereby reducing its amount in nematodes and ultimately mitigating its toxicity [25]. To verify the Cd-chelating ability of EGCG, we analyzed its UV–vis spectra in the presence of metal ions. The results showed that the UV–vis spectral curve of the EGCG solution in M9 buffer containing Cd overlapped almost entirely with the control. In contrast, the addition of Cu to the EGCG solution caused a significant change in the spectral curve, serving as a positive control (Figure 2A). In addition, the intensity and position of the maximum absorption peak in the UV–visible spectrum of EGCG treated with Cd did not change after filtration through the microporous filter membrane. Moreover, upon the addition of Cu, although the position of the maximum absorption peak remained unchanged, the intensity of the absorption peak was significantly reduced (Figure S2A). Furthermore, we used HPLC to determine the changes in the content of free EGCG after the addition of metal ions. We found that the addition of varying concentrations of Cd to the EGCG solution did not significantly alter the free EGCG content, whereas the addition of Cu resulted in a notable reduction in the free EGCG content (Figure S2B  and Figure 2B). These findings imply that EGCG does not chelate with Cd.

To investigate whether EGCG affects Cd accumulation and excretion in nematodes, we determined the Cd content in nematodes using ICP-MS. No significant change was observed in the Cd content of L1 larvae treated with EGCG for either 48 or 72 h (Figure 2C). To determine the effect of EGCG on Cd efflux in nematodes, we exposed L1 larvae to 100 μM Cd for 48 h and then treated them with EGCG for 24 or 48 h. The results showed no significant difference in Cd levels in nematodes between the EGCG-treated and control groups, regardless of whether EGCG treatment was performed for 24 or 48 h (Figure 2D), suggesting that EGCG does not affect Cd accumulation and efflux in nematodes.

Finally, we investigated the effect of non-simultaneous exposures to Cd and EGCG on nematode body size development and lifespan. Following a 2 d Cd exposure and a subsequent 2 d EGCG intervention, EGCG was found to partially reverse the Cd-induced reduction in nematode body size (Figure S2C and Figure 2E), comparable to the results obtained with simultaneous Cd and EGCG exposure (Figure 1A,B). Furthermore, EGCG was found to prolong the lifespan of nematodes exposed to Cd for 5 days and a subsequent EGCG treatment (Figure 2F). In summary, the observed reduction in Cd-induced developmental toxicity by EGCG was not achieved through either Cd chelation or reduced Cd accumulation.

### 2.3. EGCG Regulates ER Stress under Cd Exposure

**Figure 3:**
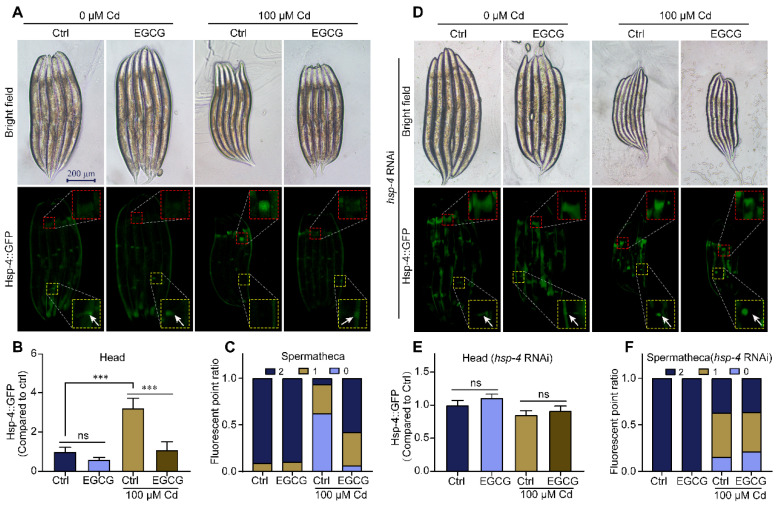

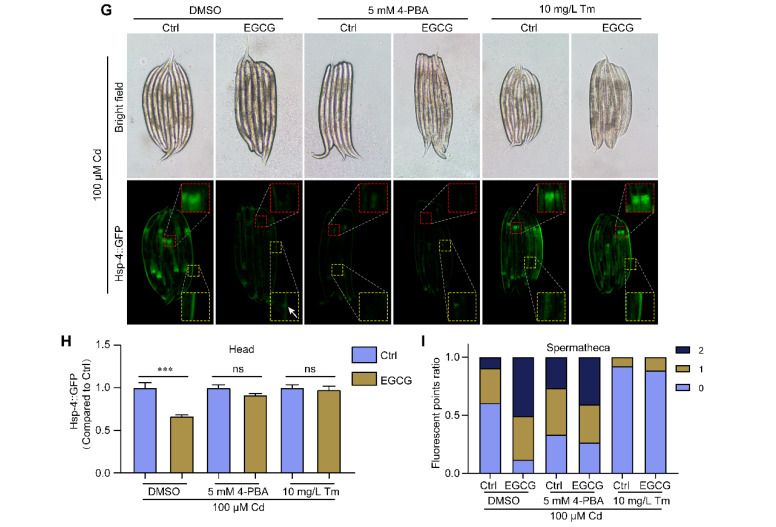
EGCG regulates Cd-induced ER stress. (**A**) Representative images of *hsp-4p::GFP*. (**B**) Relative fluorescence density in the head of *hsp-4p::GFP* animals. Relative fluorescence density was normalized to control without Cd or EGCG treatment. (**C**) Fluorescent point ratio of the spermathecae of *hsp-4p::GFP* animals. (**D**) Fluorescent micrographs of *hsp-4p::GFP* worms grown on *hsp-4* RNAi. (**E**) Relative fluorescence density of the head region in *hsp-4p::GFP* worms grown under *hsp-4* RNAi. Relative fluorescence density was normalized to control without Cd or EGCG treatment. (**F**) Ratio of fluorescence dots in the spermathecae of *hsp-4p::GFP* worms grown under *hsp-4* RNAi. (**G**) Fluorescent micrographs of *hsp-4p::GFP* worms supplemented with DMSO, 5 mM 4-PBA, or 10 mg/L Tm. (**H**) Relative fluorescence density of the head region in *hsp-4p::GFP* worms supplemented with DMSO, 5 mM 4-PBA, or 10 mg/L Tm. Relative fluorescence density was normalized to control without EGCG treatment. (**I**) Ratio of fluorescence dots in the spermathecae of *hsp-4p::GFP* worms supplemented with DMSO, 5 mM 4-PBA, or 10 mg/L Tm. L1-stage nematodes treated with Cd (0 or 100 μM) and EGCG (0 or 400 μM) for 3 d. White arrows indicate fluorescent dots in the spermathecae region. One-way ANOVA and Tukey post hoc tests were used to assess significance: *** *p* < 0.001, ns = no significance.

Cd induces ER stress and activates the endoplasmic reticulum unfolded protein response (UPR^ER^) [26]. To test the effect of EGCG on ER stress under Cd exposure, we used the *hsp-4p::GFP* strain as a reporter gene [27]. While the overall expression of *hsp-4p::GFP* was minimally affected by Cd and EGCG (Figure 3A), there was a significant difference in its expression in the head region of the nematode. Additionally, Marcella Calfon et al. showed that in the absence of drug treatment, there is constitutive *hsp-4p::GFP* expression in both spermathecae (Figure S3A). In a Cd-free environment, EGCG had little effect on the expression of *hsp-4p::GFP*. However, exposure to Cd induced *hsp-4p::GFP* expression in the nematode head and suppressed expression in the spermathecae. Supplementation with EGCG significantly suppressed the expression of the *hsp-4p::GFP* in the nematode head and appeared to partially restore the expression in the spermathecae (Figure 3A–C). Interestingly, *hsp-4* RNA interference (RNAi) induced the expression of *hsp-4p::GFP* (Figure 3D). Furthermore, when subjected to *hsp-4* RNAi, Cd was unable to induce ER stress in the nematode head, and EGCG lost its ability to inhibit ER stress in the nematode head and increase the expression in the spermathecae (Figure 3D–F).

To further confirm the modulatory effect of EGCG on ER stress under Cd exposure, we treated *hsp-4p::GFP* nematodes with 4-phenylbutyric acid (4-PBA), which inhibits ER stress by accelerating protein folding. Our findings indicated that even after supplementation with 4-PBA in a Cd-free environment, EGCG still had no effect on the expression of *hsp-4p::GFP* in the head or spermathecae (Figure S3B–D). Under Cd exposure supplemented with 4-PBA, we observed no significant difference in ER stress between the EGCG-treated and control groups, either in the head or in the spermathecae (Figure 3G–I). In addition, we treated the nematodes with tunicamycin (Tm), an ER stress inducer. We observed a significant increase in *hsp-4p::GFP* expression in the head after supplementation with Tm in a Cd-free environment, and this increase could not be suppressed by EGCG. Furthermore, the expression in the spermathecae was significantly reduced, with no significant difference observed between the EGCG-treated and control groups (Figure S3B–D). Under Cd exposure supplemented with Tm, no significant difference in the expression of *hsp-4p::GFP* in the head was observed between the EGCG-treated and control groups; the same was true for the expression in the spermathecae (Figure 3G–I). Overall, our data suggest that EGCG has a differential effect on ER stress in nematodes under Cd exposure, inhibiting ER stress in the head region and inducing ER stress in the spermathecae.

### 2.4. EGCG Attenuates Cd-Induced Body Size Developmental Toxicity by Regulating ER Stress

**Figure 4:**
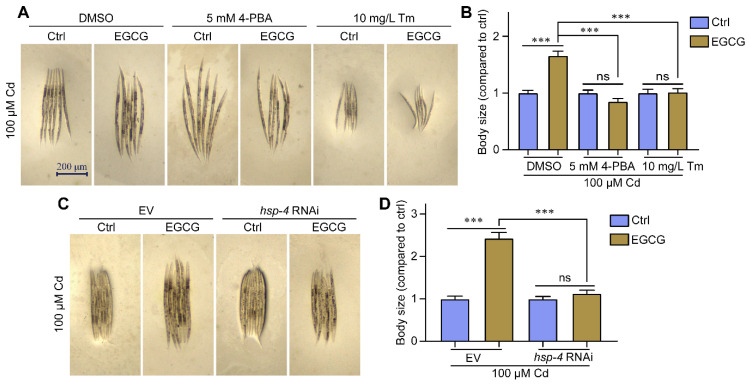
EGCG attenuates Cd-induced body size developmental toxicity by regulating ER stress. (**A**) Micrographs of WT worms supplemented with DMSO, 5 mM 4-PBA, or 10 mg/L Tm. (**B**) Body size of WT worms supplemented with DMSO, 5 mM 4-PBA, or 10 mg/L Tm. Body size was normalized to control without EGCG treatment. (**C**) Micrographs of WT worms grown on EV or *hsp-4* RNAi; (**D**) body size of WT worms grown on EV or *hsp-4* RNAi. Body size was normalized to control without EGCG treatment. L1-stage nematodes treated with Cd (100 μM) and EGCG (0 or 400 μM) for 3 d. One-way ANOVA and Tukey post hoc tests were used to assess significance: *** *p* < 0.001, ns = no significance.

EGCG was shown to attenuate Cd-induced body size developmental toxicity as well as regulate ER stress (Figure 1A,B and Figure 3A–C). To confirm whether EGCG attenuates body size developmental toxicity by modulating ER stress, we measured nematode body size under various ER stress inhibitors and activators. In a Cd-free environment, EGCG failed to increase nematode body size upon supplementation with 5 mM 4-PBA (Figure S4A,B). Under 100 μM Cd exposure, EGCG significantly increased the body size when supplemented with DMSO, but was unable to do so when supplemented with 5 mM 4-PBA (Figure 4A,B). In the absence of Cd, the addition of Tm significantly reduced the nematode body size compared to the addition of DMSO, and the addition of EGCG did not increase the nematode body size in the presence of Tm (Figure S4A,B). Under Cd exposure, the addition of Tm further reduced the nematode body size compared to the addition of DMSO, and EGCG did not restore the body size in the presence of Tm (Figure 4A,B). Dithiothreitol (DTT), another ER stress activator, showed similar results to Tm (Figure S4C).

To further verify that the attenuating effect of EGCG on Cd was related to ER stress, we used RNAi to reduce the expression of the ER stress marker HSP-4. Upon RNAi in a Cd-free environment, EGCG still had no effect on body size increase (Figure S4D,E). Under Cd exposure, EGCG partially restored nematode body size in the empty vector (EV) group, but no significant difference in body size was observed between EGCG and control treatments in the *hsp-4* RNAi group (Figure 4C,D). In conclusion, both ER inhibitors and activators, as well as *hsp-4* RNAi, abolished the EGCG-induced increase in nematode body size under Cd exposure, suggesting that EGCG reduces Cd-induced body size developmental toxicity by modulating ER stress.

### 2.5. EGCG Regulates ER Stress via the Pek-1/eif-2α/atf-4 Pathway

**Figure 5:**
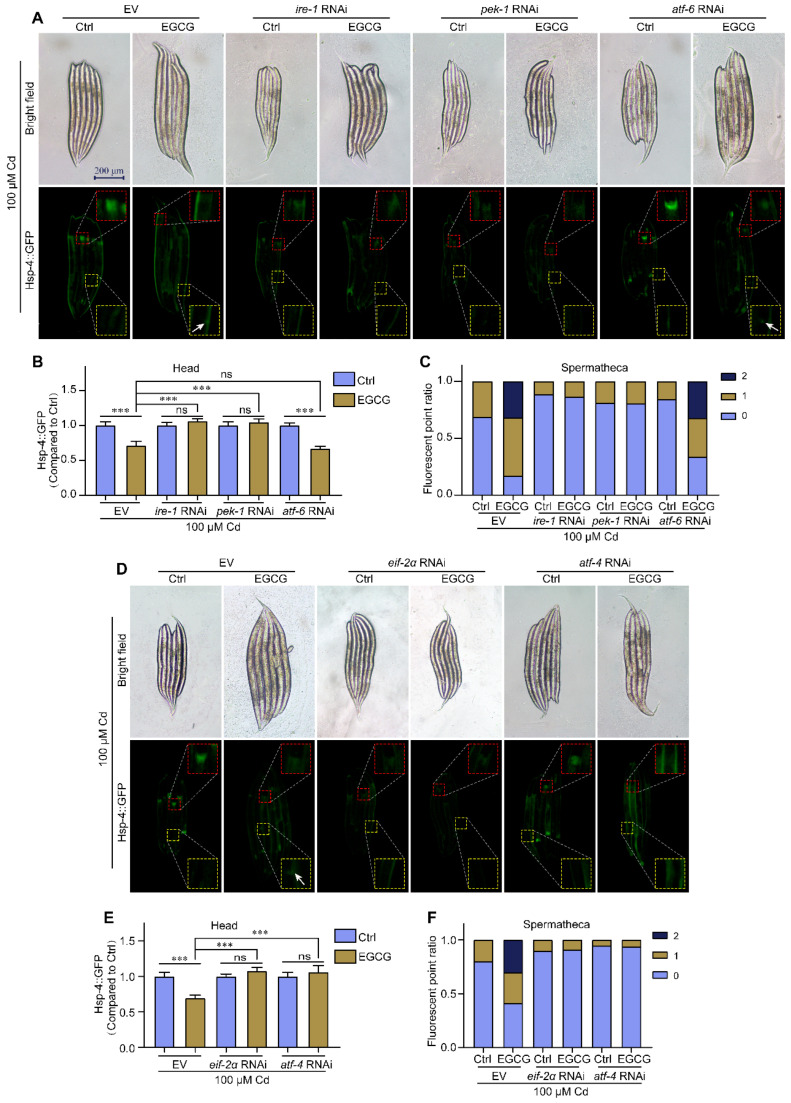
EGCG regulates Cd-induced ER stress via the *eif-2α*/*atf-4* pathway. (**A**) Fluorescent micrographs of SJ4005 worms grown under EV, *ire-1* RNAi, *pek-1* RNAi, or *atf-6* RNAi conditions. (**B**) Relative fluorescence density in the head of *hsp-4p::GFP* worms grown under EV, *ire-1* RNAi, *pek-1* RNAi, or *atf-6* RNAi. Relative fluorescence density was normalized to control without EGCG treatment. (**C**) Ratio of fluorescence dots in the spermathecae of *hsp-4p::GFP* worms grown under EV, *ire-1* RNAi, *pek-1* RNAi, or *atf-6* RNAi. (**D**) Fluorescent micrographs of *hsp-4p::GFP* worms grown under EV, *eif-2α* RNAi, or *atf-4* RNAi. (**E**) Relative fluorescence density in the head of *hsp-4p::GFP* worms grown under EV, *eif-2α* RNAi, or *atf-4* RNAi. Relative fluorescence density was normalized to control without EGCG treatment. (**F**) Ratio of fluorescence dots in the spermathecae of *hsp-4p::GFP* worms grown under EV, *eif-2α* RNAi, or *atf-4* RNAi. L1-stage nematodes treated with Cd (100 μM) and EGCG (0 or 400 μM) for 3 d. White arrows indicate fluorescent dots in the spermathecae. One-way ANOVA and Tukey post hoc tests were used to assess significance: *** *p* < 0.001, ns = no significance.

In multicellular eukaryotes, UPR^ER^ is mediated by three distinct conserved arms, namely IRE1, PERK, and ATF-6 [28]. To identify the arms through which EGCG regulates ER stress, RNAi was used to reduce the expression of *ire-1*, *pek-1,* and *atf-6* in *hsp-4p::GFP* nematodes and determine their effects on ER stress. Our results showed that, after RNAi of *ire-1*, *pek-1*, and *atf-6* in a Cd-free environment, there was no significant difference in *hsp-4p::GFP* expression in the heads of either EGCG-treated or control nematodes. Furthermore, compared to EV, RNAi of *ire-1* significantly reduced the number of spermathecae fluorescent spots, but EGCG could not change the number of fluorescent spots in the spermathecae, and RNAi of *pek-1* and *atf-6* had effects similar to EV (Figure S5A–C). Under Cd exposure, EGCG significantly reduced ER stress in the nematode head and increased the brightness and number of spermathecae fluorescent spots in the EV group but failed to do so in the *ire-1* RNAi group. Similar to the *ire-1* RNAi group, the *pek-1* RNAi group did not respond to EGCG treatment. However, in the *atf-6* RNAi group, EGCG significantly reduced ER stress in the nematode head and increased the brightness and number of spermathecae fluorescent spots, similar to the EV group (Figure 5A–C). These results suggest that EGCG regulates Cd-induced ER stress via *ire-1* and *pek-1,* but not via *atf-6*.

*Eif-2α* and *atf-4* are found downstream of PERK, and these genes are homologous to nematode *eif-2α*, *atf-4*, and *pek-1*, respectively. In the Cd-free environment, EGCG treatment in the *eif-2α* RNAi group slightly increased *hsp-4p::GFP* expression in the head but had no effect on expression in the spermathecae, while in the *atf-4* RNAi group, EGCG treatment caused no significant difference in *hsp-4p::GFP* expression in the head and spermathecae (Figure S5D–F). Under Cd exposure, EGCG did not affect *hsp-4p::GFP* expression in the head and spermathecae in the *eif-2α* RNAi and *atf-4* RNAi groups compared to the control (Figure 5D–F). Overall, these results suggest that EGCG regulates Cd-induced ER stress through the *pek-1*/*eif-2α*/*atf-4* pathway.

### 2.6. EGCG Reduces Cd-Induced Body Size Developmental Toxicity in Nematodes via the Pek-1/eif-2α/atf-4 Pathway

**Figure 6:**
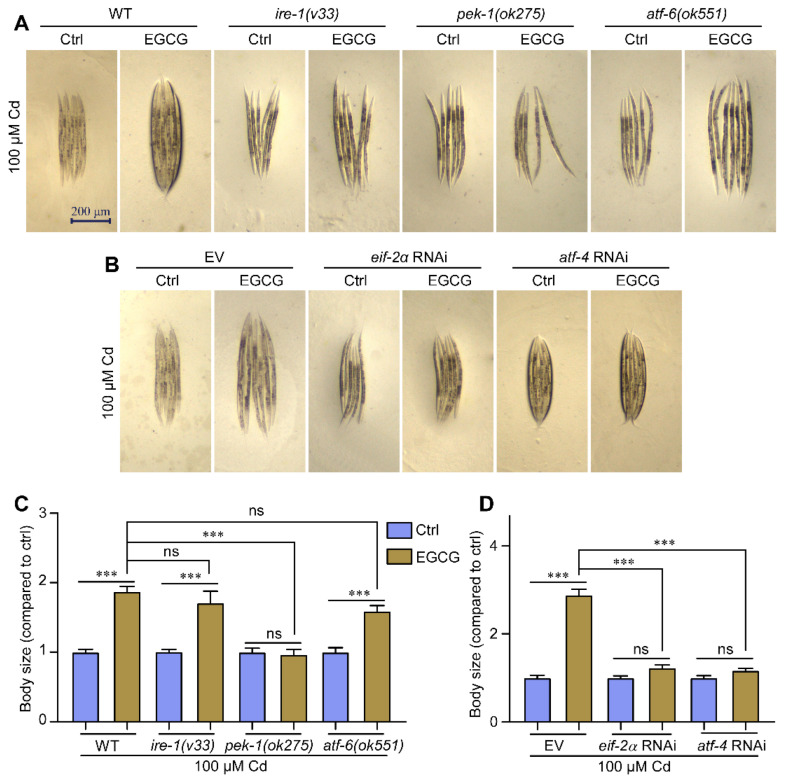
EGCG attenuates Cd-induced body size developmental toxicity via the *pek-1*/*eif-2α*/*atf-4* pathway. (**A**) Micrographs of WT, *ire-1 (v33)*, *pek-1 (ok275)*, and *atf-6 (ok551)* nematodes. (**B**) Micrographs of WT worms grown under EV, *eif-2α* RNAi, or *atf-4* RNAi. (**C**) Body size of WT, *ire-1 (v33)*, *pek-1 (ok275)*, and *atf-6 (ok551)* nematodes. Body size was normalized to control without EGCG treatment. (**D**) Body size of WT worms grown under EV, *eif-2α* RNAi, or *atf-4* RNAi. Body size was normalized to control without EGCG treatment. L1-stage nematodes treated with Cd (100 μM) and EGCG (0 or 400 μM) for 3 d. One-way ANOVA and Tukey post hoc tests were used to assess significance: *** *p* < 0.001, ns = no significance.

Using mutant strains of the three transmembrane proteins (IRE1, PERK, and ATF6) involved in the UPR^ER^, we investigated the effect of EGCG on the body size of each mutant strain to determine its impact on Cd-induced developmental toxicity. In a Cd-free environment, EGCG did not affect the body size of *ire-1 (v33)*, *pek-1 (ok275)*, and *atf-6 (ok551)* nematodes, similar to WT nematodes (Figure S6A,C). Under Cd exposure, EGCG significantly increased the body size of WT, *ire-1 (v33),* and *atf-6 (ok551)* nematodes, but not *pek-1 (ok275)* nematodes (Figure 6A,C). The detoxification of Cd-induced body size developmental toxicity in nematodes by EGCG was shown to be associated with *pek-1*, but not with *ire-1* and *atf-6*.

To further test whether EGCG reduces Cd-induced body size developmental toxicity via the *pek-1* branch, we performed RNAi targeting *eif-2α* and *atf-4* genes and measured the body size of the nematodes. Our data showed that in a Cd-free environment, EGCG did not affect nematode body size under *eif-2α* and *atf-4* RNAi, similar to what was observed with EV control (Figure S6B,D). Under Cd exposure, EGCG significantly increased the nematode body size under EV conditions but not under *eif-2α* RNAi. In contrast, in the *atf-4* RNAi group, EGCG slightly increased the nematode body size, but the increase was significantly less than that in the EV group (Figure 6B,D). These results suggest that EGCG reduces the body size developmental toxicity of Cd-induced nematodes via the *pek-1*/*eif-2α*/*atf-4* pathway.

## 3. Discussion

In this research we found that EGCG reduced Cd-induced body size developmental toxicity in nematodes, and this detoxification effect was independent of the chelation activity of EGCG and was not related to Cd accumulation and efflux. We also found that EGCG reduces Cd-induced body size developmental toxicity in nematodes via the PEK-1/eIF-2α/ATF-4 branch of ER stress.

The development of *C. elegans* is very sensitive to Cd exposure. Popham et al. [29] and Hirota K et al. [9] investigated the effect of Cd exposure on nematode length in NGM medium and found that the length of nematodes exposed to 100 μM Cd for 3 days was shortened by 20–30% compared to the control group. Duran-Izquierdo et al. [30] found that the length of L1 larvae exposed to 100 μM Cd in K-medium for 3 days showed a reduction in body length of about 30% compared to the control group. These findings are consistent with the results of this work (Figure 1A,B).

Some naturally occurring bioactive compounds can be used to prevent adverse health effects from Cd exposure [1]. In *C. elegans*, N-acetylcysteine significantly reversed Cd-induced aversive behavior and lifespan shortening [9]; hydroalcoholic extract of Haematoxylum brasiletto significantly reduced the lethal effect of Cd on nematodes and ameliorated Cd-induced locomotor inhibition and body length shortening [30]; selenium was found to reduce Cd-induced lethality [31].

As a natural antioxidant, EGCG is considered an attractive candidate for inhibiting Cd toxicity. Abib et al. demonstrated that EGCG protects against Cd-induced mitochondrial dysfunction in vitro [32]. EGCG increased cell viability by inhibiting Cd-induced apoptosis in HL-7702 and PC12 cells [19,20]. EGCG attenuated Cd-induced chronic kidney injury and fibrosis in male adult rats [18]. However, the effect of EGCG on cell viability under Cd exposure remains controversial. Yu et al. found that EGCG decreased PC-3 cell viability under Cd exposure [33]. Using the living organism *C. elegans* as a model, we investigated the effect of EGCG on nematode development under Cd exposure for the first time. The results showed that under Cd exposure, EGCG promoted the development of nematode body size, improved nematode developmental arrest, and partially restored the time of first egg laying (Figure 1A–E). The pharyngeal pumping rate is related to the amount of food available to C. elegans. Our finding that EGCG did not affect the pharyngeal pumping rate under Cd exposure suggests that EGCG does not ameliorate Cd-induced developmental toxicity by diet (Figure 1F). In conclusion, our results fully demonstrate the beneficial effect of EGCG on Cd-induced nematode developmental toxicity, providing strong evidence for the detoxification effect of EGCG on Cd in multicellular animals.

EGCG can chelate the heavy metal Cd, resulting in the formation of complexes that significantly reduce free Cd levels [24,34]. Abib et al. demonstrated that the pH of the environment can affect the chelation of Cd by EGCG [32]. The Cd-EGCG complex was formed at pH 8.3, but its stability decreased significantly at pH 7.6 [32], and it dissociated completely at pH 7.0 [19]. In the present study, our UV–Vis spectroscopy results showed that EGCG did not chelate with Cd in M9 buffer (pH = 7.4), but did chelate with the positive control Cu (Figure 2A and Figure S2A). This conclusion was further supported by the results of the HPLC free-EGCG assay (Figure 2B and Figure S2B).

The accumulation of heavy metals is an important cause of their toxicity, and one of the detoxification mechanisms of EGCG is its ability to reduce the accumulation of heavy metals [35]. EGCG inhibits the uptake of Cd by PC-3 cells [33] and reduces the accumulation of Cd in various tissues, including the serum, kidney, and liver, in rats [36]. However, our analysis of the Cd content in nematodes by ICP-MS showed that EGCG did not affect Cd accumulation or excretion by nematodes (Figure 2C,D). To further verify our conclusion, we intervened first with Cd and then with EGCG in nematodes and also found that EGCG reduced Cd-induced body size developmental toxicity and prolonged life span under Cd exposure (Figure 2E,F). In conclusion, our results strongly suggest that the EGCG-mediated detoxification of Cd-induced body size developmental toxicity is neither achieved through chelation, nor is it related to Cd accumulation and efflux.

EGCG has been shown to inhibit Cd-induced apoptosis in HL-7702 cells by scavenging ROS [19], and to reduce Cd-induced kidney injury in adult male rats by scavenging ROS and regulating microRNA levels [18]. Our study suggests that the beneficial effects of EGCG on Cd-induced body size developmental toxicity may be associated with the modulation of ER stress. A three-pronged test was carried out to prove this conclusion. Firstly, we measured the effect of EGCG on Cd-induced ER stress using the nematode strain SJ4005, which expresses GFP fused to the ER stress marker protein HSP-4, and showed that EGCG inhibited Cd-induced ER stress in the head region of nematodes (Figure 3A,B). Secondly, we used RNAi to reduce the expression of HSP-4 and found that it abolished the inhibitory effect of EGCG on Cd-induced ER stress in the nematode head as well as its detoxification effect on developmental toxicity (Figure 3D,E and Figure 4C,D). Finally, we supplemented the nematodes with either an ER stress inhibitor (4-PBA) or activator (Tm or DTT) and found that these supplements abolished the inhibitory effect of Cd-induced ER stress in the nematode head and the detoxification of developmental toxicity (Figure 3G,H and Figure 4A,B). In conclusion, our results provide strong evidence that EGCG-mediated detoxification of Cd-induced body size developmental toxicity is related to ER stress.

Interestingly, our data also indicate that EGCG increases ER stress in the nematode spermatheca under Cd exposure (Figure 3A,C). As previously reported, ER stress is a double-edged sword, as it activates the UPR^ER^ to restore cellular homeostasis on the one hand, but on the other hand, prolonged unresolved ER stress induces programmed cell death [37]. The exact mechanism by which EGCG increases ER stress in the spermatheca is not yet clear, as it is unclear whether EGCG induces or inhibits programmed cell death. Further studies are needed to clarify this phenomenon. In addition, we found that *hsp-4* RNAi induced the expression of *hsp-4p::GFP* (Figure 3D). Kapulkin et al. [38] also found that RNA interference with ER molecular chaperones such as *hsp-4*, *hsp-3*, etc., induced the expression of *hsp-4p::GFP* in SJ4005(zcIs4 [*hsp-4p::GFP*] V). We hypothesize that there is compensatory transcriptional regulation between ER molecular chaperones in *C. elegans* and that *hsp-4* expression is an autoregulatory loop.

The UPR^ER^ is the cellular response to ER stress to restore ER homeostasis. It consists of three branches, each of which transduces ER stress signals to the cytoplasm via transmembrane regulators (IRE1, PERK, or ATF6), thereby activating different downstream signaling pathways [26]. EGCG has beneficial effects in attenuating the toxic effects of methyl parathion on oocytes, and these effects appear to be associated with the downregulation of XBP1 downstream of IRE1 [17]. Our study showed that RNAi targeting *ire-1* abolished the inhibitory effect of EGCG on Cd-induced ER stress in the nematode head (Figure 5A,B), suggesting that the beneficial effect of EGCG is associated with IRE-1. Interestingly, however, RNAi targeting *ire-1* did not alter the detoxification effect of EGCG on Cd-induced developmental toxicity (Figure 6A,C), suggesting that EGCG does not reduce Cd-induced body size developmental toxicity via IRE-1.

PERK (PEK-1 in *C. elegans*), the second ER stress sensor, is an ER transmembrane protein [27]. In a cellular model, EGCG inhibited the expression of the ER stress marker ATF4 in trabecular meshwork cells treated with Tm [39], suppressed the increase in p-PERK/p-eIF-2α/ATF-4 expression levels in Caco-2 cells treated with Tm and thapsigargin [40], and decreased the expression of p-PERK in high-glucose-induced podocytes [41]. Our study shows that RNAi targeting *pek-1* abolishes the inhibitory effect of EGCG on Cd-induced ER stress in the nematode head (Figure 5A,B). Loss of function of PERK leads to abnormal development in patients [42], whereas RNAi targeting *eif-2α* leads to smaller body size in nematodes (Figure S6B), suggesting that PERK is associated with development. The beneficial effect of EGCG on nematode body size development under Cd exposure was abolished when *pek-1* was targeted using RNAi. These results suggest that the beneficial effects of EGCG on both Cd-induced ER stress and body size developmental toxicity are associated with PERK. To further support this conclusion, we downregulated the expression of PERK downstream genes *eif-2α* and *atf-4* using RNAi. It was found that RNAi targeting *eif-2α* and *atf-4* also reduced both the regulatory effect of EGCG on ER stress (Figure 5C,D) and the alleviation of body size developmental toxicity (Figure 6B,D). In conclusion, the beneficial influence of EGCG on Cd-induced body size developmental toxicity was associated with the PERK-ATF4 pathway.

Finally, the detoxifying effect of EGCG on Cd-induced body size developmental toxicity remained unaffected by the RNAi of *atf-6* (Figure 6A,C). In summary, our results suggest that the mitigating effect of EGCG on Cd-induced nematode body size developmental toxicity is associated with the PERK branch, but not with IRE1 and ATF6. However, our study solely established the beneficial effects of EGCG on body size developmental toxicity in nematodes and performed no further validation in higher animals.

## 4. Materials and Methods

### 4.1. C. elegans Strains and Maintenance

The N2, RB772 (*atf-6(ok551) X*), RE666 (*ire-1(v33) II*), RB545 (*pek-1(ok275) X*), and SJ4005 (*zcIs4* [*hsp-4p::GFP*] *V*) strains were obtained from the *C. elegans* Genetics Center (CGC; University of Minnesota, MN, USA). Except for during the RNA interference (RNAi) experiments, strains were cultured on standard nematode growth medium (NGM) containing *Escherichia coli* OP50 and maintained at 20 °C and 50% relative humidity. Synchronized nematodes were obtained using the bleaching method, which involved isolating eggs from gravid adults and treating them with bleach to remove any contamination [43].

### 4.2. C. elegans Food

The *E. coli* OP50 monoclonal culture was incubated in Luria broth (LB) for 12 h and then expanded for an additional 4.5 h. The concentrated *E. coli* were resuspended in M9 medium and coated on NGM dishes for use in the experiment.

### 4.3. Drugs and Treatment

EGCG (purity > 98%) was supplied by Chengdu Wagott Bio-tech Co., Ltd. (Chengdu, China). CdCl_2_, 4-phenylbutyric acid (4-PBA), and tunicamycin (Tm) were supplied by Shanghai Aladdin Biochemical Technology Co. (Shanghai, China). Cd in the form of CdCl_2_ was added to NGM medium to a final concentration of 100 μM prior to coagulation, and synchronized L1 larvae were added to NGM medium containing 100 μM Cd to start the intervention. EGCG was added to *E. coli* OP50 feed to a final concentration of 400 μm and coated onto NGM solid petri dishes. Unless otherwise stated, EGCG and Cd were treated simultaneously on L1 larvae and indices were determined after 3 d of treatment. 4-PBA and Tm were added to the concentrated E. coli OP50 solution prior to coating.

### 4.4. C. elegans Body Size Estimation

Synchronized L1 larvae were transferred to NGM petri dishes containing 0 μM or 100 μM Cd (0 μM or 400 μM EGCG in the diet) and incubated for 3 d. The images of the treated nematodes were obtained at 50× magnification under a stereomicroscope, and the nematode body length and width were measured using ImageJ V1.8.0.112 software [44]. Body size was estimated from body length and width using the following formula:

(1) $$Body\;size=\pi \times {\left(\frac{Body\;width}{2}\right)}^{2}\times Body\;length$$

### 4.5. Analysis of C. elegans Development

*C. elegans* embryos hatch with 558 nuclei and become a first-stage larva (L1). The animal begins feeding and develops through four larval stages (L1–L4). L4 larvae continue to develop for about 12 h before starting to produce offspring that become adults [22]. Synchronized animals were added to NGM plates containing drugs and cultured for 48 h. Subsequently, the number of animals at various stages of development (L1/L2, L3, and L4/adult) were quantified, with a minimum of three biological replicates performed for each group.

### 4.6. Onset of Egg Laying

To examine the reproductive development of *C. elegans*, synchronized L1 larvae were transferred to experimental plates containing different drugs. After 48 h, 8–10 nematodes were picked from each experimental plate and transferred to the corresponding fresh plates. The egg-laying status of these nematodes was observed every hour, and the time to first egg laying was recorded.

### 4.7. Lifespan Experiments

The lifespan of nematodes was measured using the methods of Sutphin et al. [45]. Briefly, on day 0, synchronized L1 larvae were added to NGM plates containing different drugs and cultured. After 3 d, approximately 35 nematodes were randomly selected from each dish and transferred to NGM dishes containing 5′-fluorodeoxyuridine (50 μM) without drug. The number of surviving individuals was counted every 2 d, and mortality was calculated until all individuals had died. All lifespan experiments were conducted in a double-blind manner. 

### 4.8. Pharyngeal Pumping Rate

The pharyngeal pump rate was measured 3 d after intervention on L1 larvae. The number of pharyngeal pump contractions in nematodes was observed for 20 s with observations recorded at 10 s intervals, and this process was repeated three times in succession. Eight to ten nematodes were randomly measured per treatment, and each treatment was replicated three times.

### 4.9. UV–vis Spectra of EGCG

EGCG was dissolved in M9 buffer to prepare a 400 μM EGCG solution, to which 100 μM or 400 μM CdCl_2_ was added subsequently. CuSO_4_ was used as a positive control. After 4 h, the UV–vis absorption spectra of EGCG were measured using the Shimadzu UV2600 UV–vis spectrophotometer (Shimadzu, Kyoto, Japan) at 20 °C. The samples were filtered through a 0.22 μm pore size microporous membrane before being subjected to UV–vis spectroscopy.

### 4.10. Free EGCG Content Assay

Free EGCG was measured by the method of Midori Yasuda et al. [46] using high-performance liquid chromatography (HPLC, LC-20AT, Shimadzu). The mobile phase consisted of water (A) and methanol (B) with the addition of 0.2% methanoic acid. The volume, flow rate, column, temperature, and pressure were 10 μL, 1.0 mL/min, 40 °C, and 13.69 MPa, respectively.

### 4.11. Accumulation and Efflux of Cd in Nematodes

To obtain sufficient numbers of nematodes, culture was carried out using 9 cm Petri dishes. To prevent food deprivation, nematodes were transferred daily to new NGM Petri dishes. Cd accumulation was determined by culturing a sufficient number of synchronized L1 nematodes in separate media containing 100 μM Cd and 100 μM Cd + 400 μM NGM, and nematodes were collected after 48 and 72 h of incubation. Cd efflux was determined by culturing a sufficient number of synchronized L1 nematodes in NGM medium containing 100 μM Cd for 48 h, which were then transferred to separate media containing 0 μM EGCG and 400 μM EGCG, and nematodes were collected after 24 and 48 h of incubation. All collected nematodes were resuspended 5 times in M9 buffer containing 10% Tween and once in sterile water to remove residual *E. coli* OP50, and then frozen several times in liquid nitrogen before being sonicated and crushed. A BCA method was used to quantify protein in the supernatant of the crushed extract after centrifugation. The remaining supernatant and precipitate were added to an equal volume of nitric acid and digested at 95 °C for at least 12 h. The Cd content of the solution was determined using inductively coupled plasma mass spectrometry (ICP-MS) (Nexion 300X, PerkinElmer Corporation, Waltham, MA, USA), according to the standard method.

### 4.12. RNAi

*E. coli* HT115 (DE3) expressing homologous dsRNA was used to generate loss-of-function RNAi phenotypes [47]. A vector containing HT115 was grown overnight at 37 °C in LB medium containing ampicillin (100 mg/L). After inducing the culture with IPTG for 3 h at 37 °C, the bacterial solution was plated on NGM plates containing ampicillin and streptomycin.

### 4.13. Determination of hsp-4p::GFP Expression Levels

The nematodes were placed on agar coverslips coated with a 2% agarose solution, and 3–5 µL of a 0.2 mM levamisole solution was added to induce paralysis. Fluorescence images were captured using a Nikon ECLIPSE Ti-U fluorescence microscope (Nikon Group, Tokyo, Japan). The expression of *hsp-4p::GFP* in nematode heads was analyzed for mean fluorescence intensity using ImageJ V1.8.0.112 software. The expression of *hsp-4p::GFP* in the spermatheca was assessed by counting the number of fluorescent spots in the spermatheca and scoring as follows: 0, no fluorescent bright spots; 1, one fluorescent bright spots; 2, two fluorescent bright spots.

### 4.14. Statistical Analysis

Each treatment was replicated in 3 Petri dishes with at least 50 nematodes in each dish. GraphPad Prism 8 software was used for statistical analysis and graphing, and all results were expressed as mean ± standard error of mean (SEM). Data were tested for normality using the Shapiro–Wilk test. Student’s t-test was used for two data sets that met normality, and one-way ANOVA and Tukey post hoc tests were used for data sets with more than two data sets. Survival analysis was performed using the Kaplan–Meier test, and *p*-values were obtained using the log-rank test. *p* < 0.05 was considered statistically significant.

## 5. Conclusions

In conclusion, our study provides strong evidence that EGCG reduces Cd-induced body size developmental toxicity in nematodes by regulating the PEK-1/eIF-2α/ATF-4 pathway, delivering crucial evidence for tea detoxification.

## Data Availability

Data is contained within the article.

## Supplementary Materials

The following supporting information can be downloaded at: https://www.mdpi.com/article/10.3390/molecules28176344/s1, Effects of different concentrations of Cd and EGCG on nematode body size (Figure S1), Influence of EGCG on Cd status (Figure S2), Influence of EGCG on ER stress in a Cd-free environment (Figure S3), Impact of EGCG on nematode body size in a Cd-free environment (Figure S4), Effects of EGCG on ER stress in nematode RNAi targeting ER-related genes in a Cd-free environment (Figure S5), and Effects of EGCG on body size in nematode RNAi targeting ER-related genes in a Cd-free environment (Figure S6).
