# Supplementary material for: Epigallocatechin-3-Gallate Reduces Cd-Induced Developmental Toxicity of Bodysize in Caenorhabditis elegans via the PEK-1/eIF-2α/ATF-4 Pathway
Source: Molecules. 2023 Aug 30;28(17):6344. doi: 10.3390/molecules28176344 (PMC10489720; doi:10.3390/molecules28176344)
Supplement: Supplementary file 1 [file molecules-28-06344-s001.zip › molecules-2573696-SI.pdf]

Supplementary materials

Epigallocatechin-3-gallate Reduces Cd-induced Developmental Toxicity of Body Size in *Caenorhabditis elegans* via the PEK-1/eIF-2α/ATF-4 Pathway

Shuanghui Wang [1,2,*], Chuhong Chen [2], Yan Lu [1]

[1] National Research Center of Engineering and Technology for Utilization of Botanical Functional Ingredients from Botanicals, Hunan Agricultural University, Changsha, Hunan 410128, China

[2] Key Laboratory of Green Control of Crop Pests in Hunan Higher Education, Hunan University of Humanities, Science and Technology, Loudi, Hunan 417000; China

* Corresponding authors: 3000@huhst.edu.cn; Phone: +86-18216475975

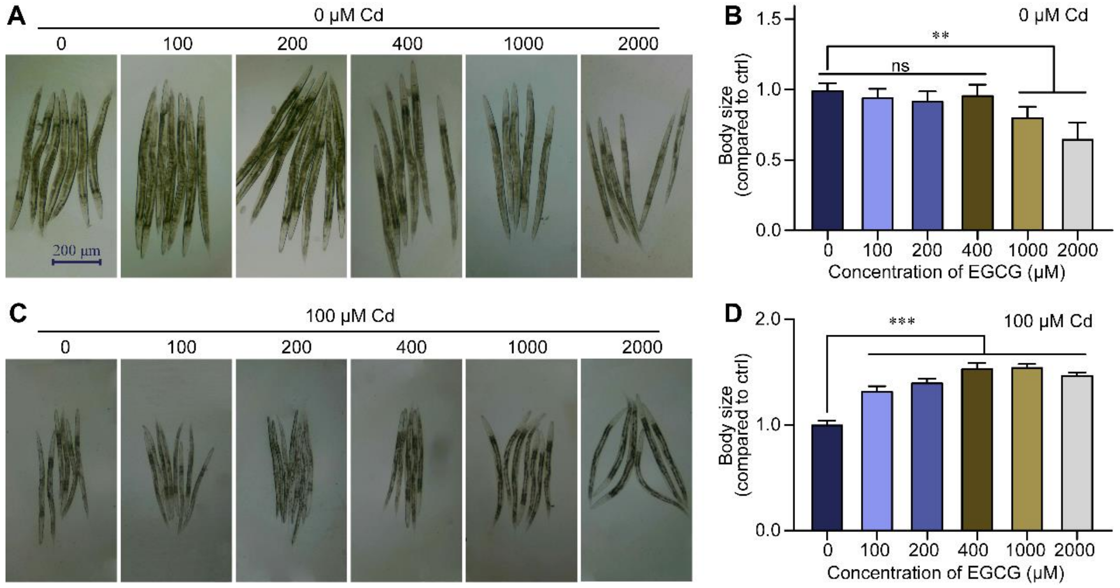

Figure S1. Effects of different concentrations of Cd and EGCG on nematode body size. (A) Micrographs of L1 nematodes treated with different EGCG concentrations for 3 d. (B) Body size of L1 nematodes treated with different EGCG concentrations for 3 d. (C) Micrographs of L1 nematodes treated with 100 μM Cd and different concentrations of EGCG for 3 d. (D) Body size of L1 nematodes treated with 100 μM Cd and different concentrations of EGCG for 3 d. Body size was normalized to control without EGCG treatment. All error bars represent SEM, and differences were considered significant at ** $p < 0.01$ and *** $p < 0.001$; ns, no significance.

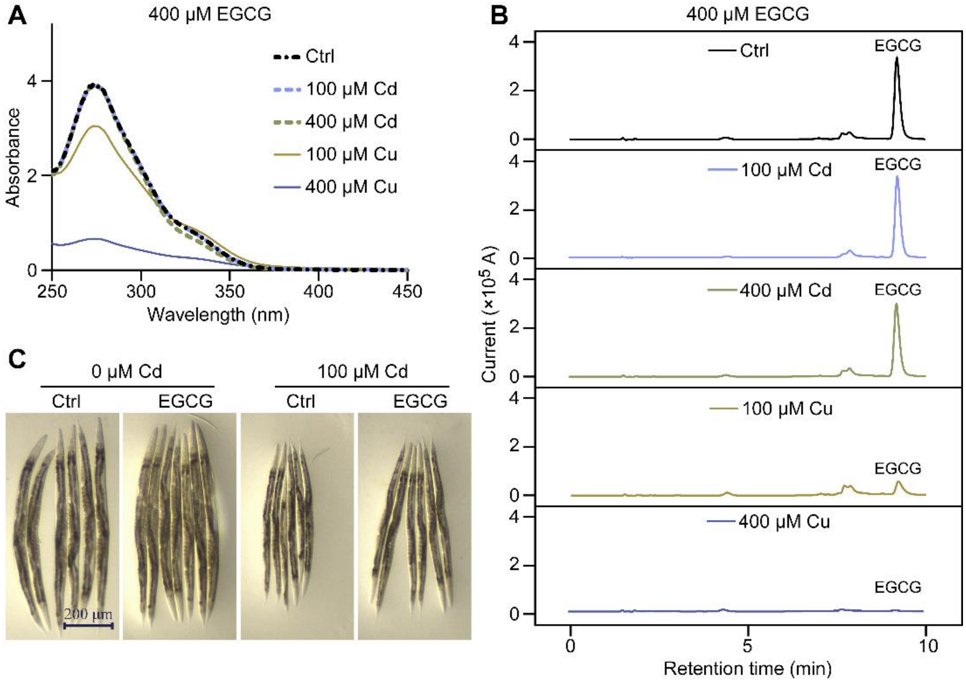

Figure S2. Influence of EGCG on Cd status. UV–Vis spectra(A) and EGCG chromatogram by HPLC (B) of 400 μM EGCG treated with 100 or 400 μM Cd and Cu for 4 h, followed by filtration through a 0.22-μm-pore-size microporous filter membrane; (C) Micrographs of L1 nematodes treated with Cd (0 or 100 μM) for 48 h followed by EGCG (0 or 400 μM) treatment for 48 h.

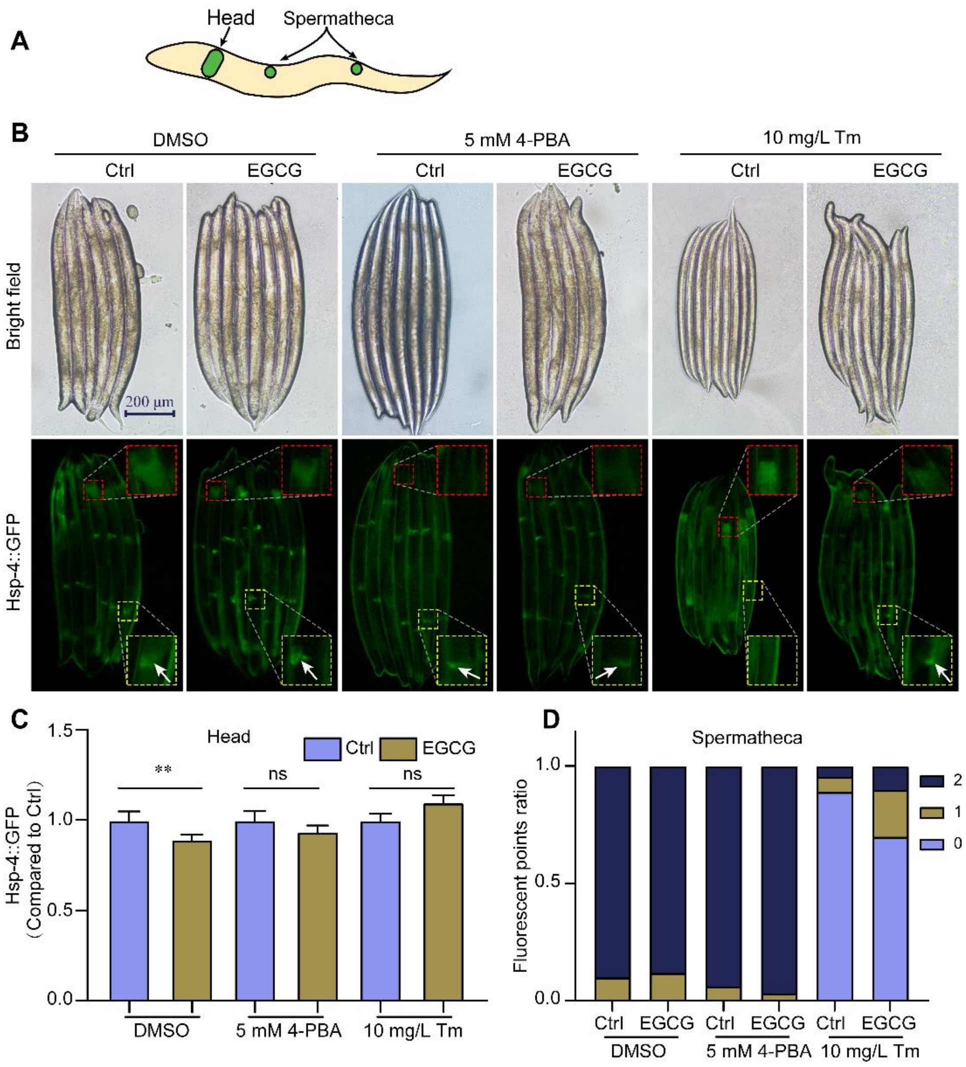

Figure S3. Influence of EGCG on ER stress in a Cd-free environment.（A）Expression mode of Hsp-4p::GFP; Fluorescent micrographs (B), relative fluorescence density in the head (C) and ratio of fluorescence dots in the spermathecae (D) of L1-stage *hsp-4p*::*GFP* worms treated with EGCG (0 or 400 μM) for 3 d and supplemented with DMSO, 5 mM 4-PBA or 10 mg/L Tm. Relative fluorescence density was normalized to control without EGCG treatment. White arrows indicate fluorescent dots on the spermathecae. All error bars represent SEM. Differences were considered significant at ** $p < 0.01$; ns, no significance.

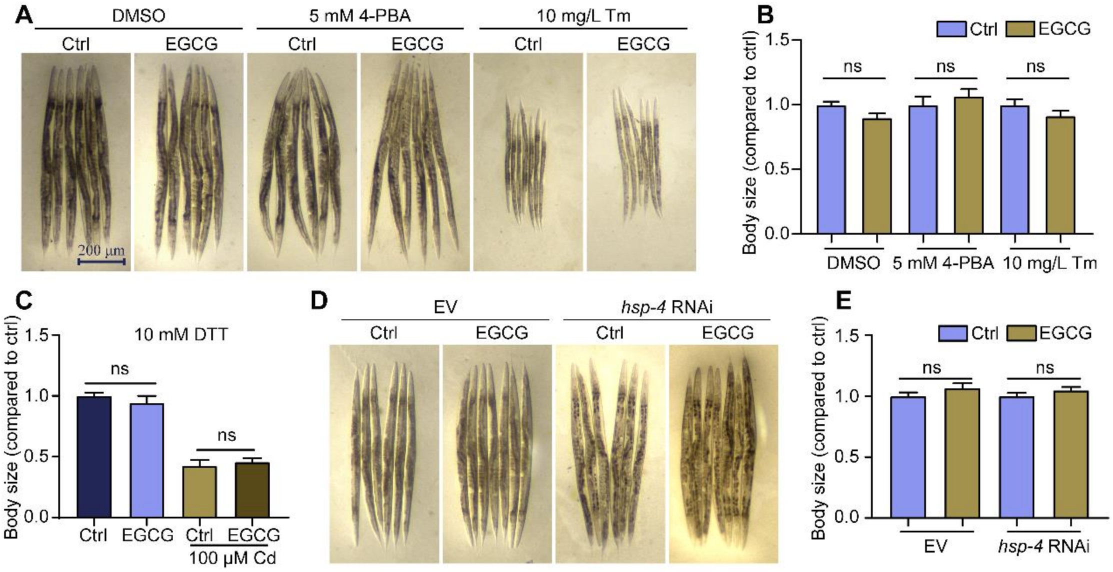

Figure S4. Impacts of EGCG on nematode body size in a Cd-free environment. (A) Micrographs of WT L1 worms treated with EGCG (0 or 400 μM) for 3 d supplemented with dimethyl sulfoxide (DMSO), 5 mM 4-PBA, or 10 mg/L Tm. (B) Body size of WT L1 worms treated with EGCG (0 or 400 μM) for 3 d supplemented with DMSO, 5 mM 4-PBA, or 10 mg/L Tm. Body size was normalized to control without EGCG treatment. (C) Body size of WT L1 worms treated with Cd (0 or 100 μM) and EGCG (0 or 400 μM) for 3 d supplemented with 10 mM DTT. Body size was normalized to the control without Cd or EGCG treatment. (D) Micrographs of WT L1 worms treated with EGCG (0 or 400 μM) for 3 d, grown on EV or *hsp-4* RNAi. (E) Body size of WT L1 worms treated with EGCG (0 or 400 μM) for 3 d, grown on EV or *hsp-4* RNAi. Body size was normalized to control without EGCG treatment. All error bars represent SEM. Differences were considered significant at * $p < 0.05$; ns, no significance.

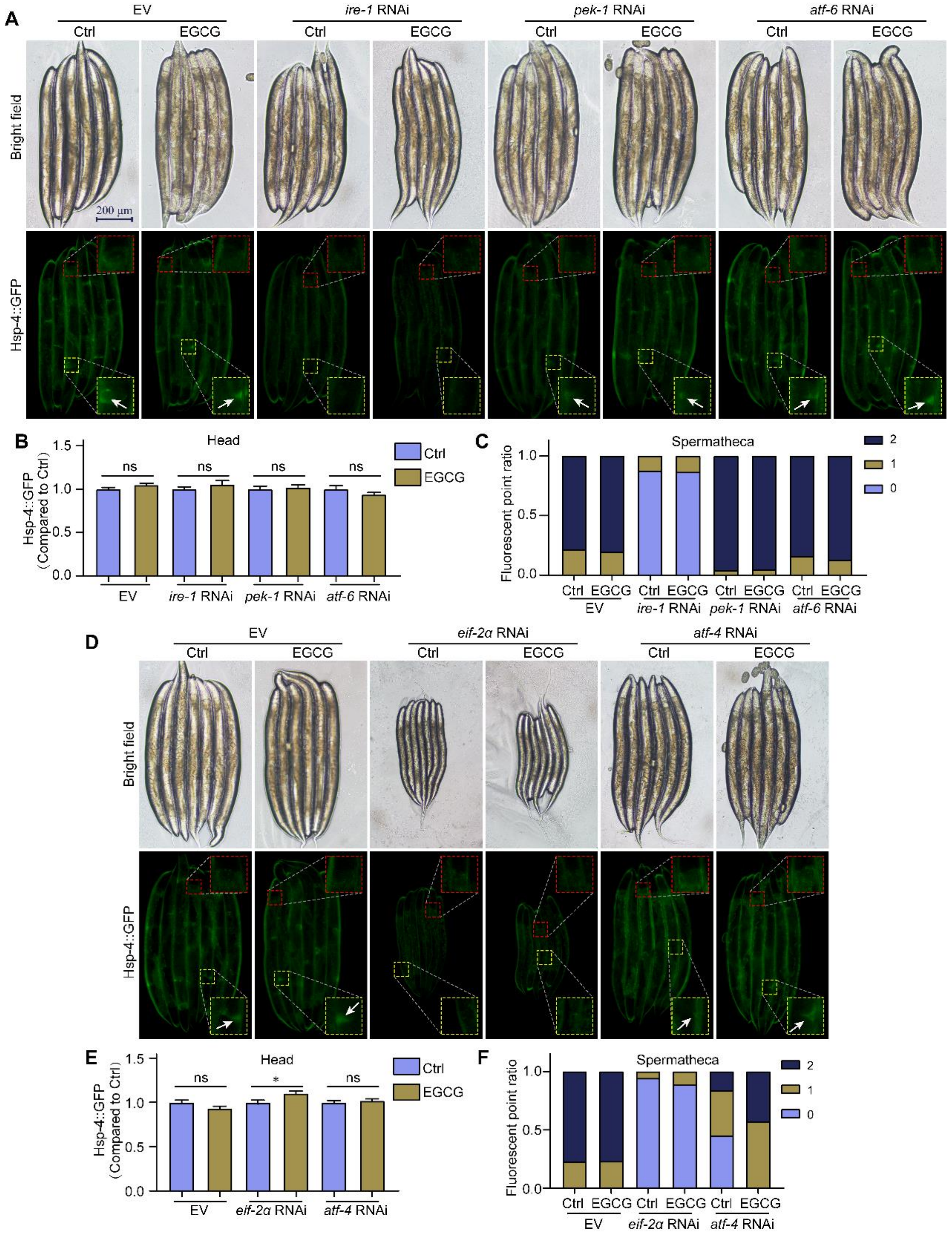

Figure S5. Effects of EGCG on ER stress in nematode RNAi targeting ER-related genes in a Cd-free environment. (A) Fluorescent micrographs of *hsp-4p::GFP* worms grown on EV, i*re-1* RNAi, *pek-1* RNAi, or *atf-6* RNAi. (B) Relative fluorescence density in the head of *hsp-4p*::*GFP* worms grown on EV, *ire-1* RNAi, *pek-1* RNAi, or *atf-6* RNAi. (C) Ratio of fluorescence dots in the spermathecae of *hsp-4p*::*GFP* worms

grown on EV, *ire-1* RNAi, *pek-1* RNAi, or *atf-6* RNAi. (D) Fluorescent micrographs of *hsp-4p*::*GFP* worms grown on EV, eif-2α RNAi, or atf-4 RNAi. (E) Relative fluorescence density in the head of *hsp-4p*::*GFP* worms grown on EV, *eif-2α* RNAi, or *atf-4* RNAi; (F) Ratio of fluorescence dots in the spermathecae of *hsp-4p*::*GFP* worms grown on EV, *eif-2α* RNAi, or *atf-4* RNAi. L1 nematodes treated with EGCG (0 or 400 μM) for 3 d. Relative fluorescence density was normalized to control without EGCG treatment. White arrows indicate fluorescent dots on the spermathecae. All error bars represent SEM. Differences were considered significant at *** $p < 0.001$; ns, no significance.

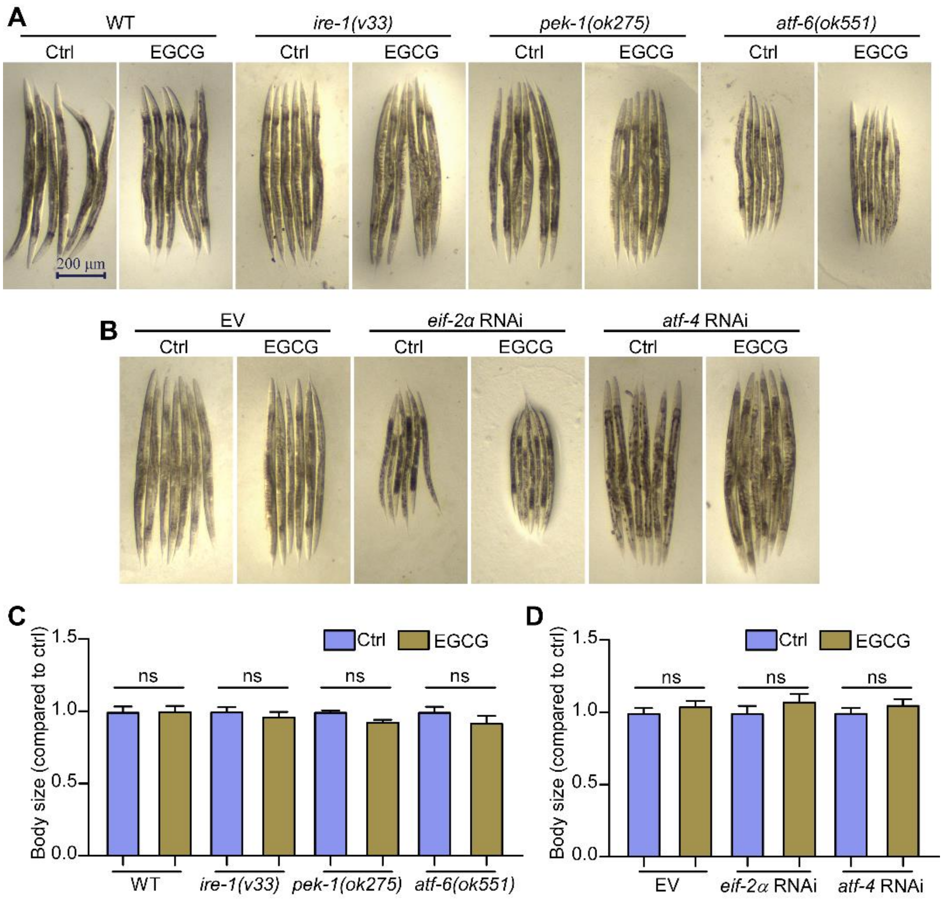

Figure S6. Effects of EGCG on body size in nematode RNAi targeting ER-related genes in a Cd-free environment. (A) Micrographs of WT, *ire-1(v33)*, *pek-1 (ok275)*, and *atf-6 (ok551)*. (B) Micrographs of WT worms grown on EV, eif-2α RNAi, or atf-4 RNAi. (C) Body size of WT, *ire-1 (v33)*, *pek-1 (ok275)*, and *atf-6 (ok551)*. (D) Body

size of WT worms grown on EV, *eif-2α* RNAi, or *atf-4* RNAi. L1 nematodes treated with EGCG (0 or 400 μM) for 3 d. Body size was normalized to control without EGCG treatment. All error bars represent SEM. Differences were considered significant at ** $p < 0.01$ and *** $p < 0.001$; ns, no significance.
